# Transcription Factors Regulation in Human Peripheral White Blood Cells during Hypobaric Hypoxia Exposure: an *in-vivo* experimental study

**DOI:** 10.1038/s41598-019-46391-6

**Published:** 2019-07-09

**Authors:** Sandro Malacrida, Alessandra Giannella, Giulio Ceolotto, Carlo Reggiani, Alessandra Vezzoli, Simona Mrakic-Sposta, Sarah Moretti, Rachel Turner, Marika Falla, Hermann Brugger, Giacomo Strapazzon

**Affiliations:** 1 Institute of Mountain Emergency Medicine, Eurac Research, Bolzano, Italy; 2 Department of Medicine-DIMED, Campus Biomedico Pietro D’Abano, University of Padova, Padova, Italy; 3 Institute of Bioimaging and Molecular Physiology, National Council of Research, Segrate (Milan), Italy; 4 Department of Biomedical Sciences, University of Padova, Padova, Italy; 5 Department of Neurology, General Hospital of Bolzano, Bolzano, Italy

**Keywords:** Transcription, Biomarkers

## Abstract

High altitude is a natural laboratory, within which the clinical study of human physiological response to hypobaric hypoxia (HH) is possible. Failure in the response results in progressive hypoxemia, inflammation and increased tissue oxidative stress (OxS). Thus, investigating temporal changes in key transcription factors (TFs) *HIF-1α*, *HIF-2α*, *NF-κB and NRF2* mRNA levels, relative to OxS and inflammatory markers, may reveal molecular targets which contrast deleterious effects of hypoxia. Biological samples and clinical data from 15 healthy participants were collected at baseline and after rapid, passive ascent to 3830 m (24 h and 72 h). Gene expression was assessed by qPCR and ROS generation was determined by EPR spectroscopy. Oxidative damage and cytokine levels were estimated by immuno or enzymatic methods. Hypoxia transiently enhanced *HIF-1α* mRNA levels over time reaching a peak after 24 h. Whereas, *HIF-2α* and *NRF2* mRNA levels increased over time. In contrast, the *NF-κB* mRNA levels remained unchanged. Plasma levels of IL-1β and IL-6 also remained within normal ranges. ROS production rate and markers of OxS damage were significantly increased over time. The analysis of TF-gene expression suggests that HIF-1α is a lead TF during sub-acute HH exposure. The prolongation of the HH exposure led to a switch between HIF-1α and HIF-2α/NRF2, suggesting the activation of new pathways. These results provide new insights regarding the temporal regulation of TFs, inflammatory state, and ROS homeostasis involved in human hypoxic response, potentially also relevant to the mediation of diseases that induce a hypoxic state.

## Introduction

The human response to hypoxia is multifactorial involving the complex synergy of hematopoietic, respiratory and cardiovascular systems to maintain adequate tissue oxygenation; failure in the response results in progressive hypoxemia, inflammation and increased tissue oxidative stress (OxS)^1^. Specifically, cellular hypoxia is known to be both a primary homeostatic moderator as well as fundamental mechanism of injury in the pathogenesis of acute diseases, but also of chronic disorders such as: atherosclerosis, arthritis, obstructive sleep apnea syndrome (OSAS) and other cardiovascular, cerebrovascular and neurodegenerative diseases^2–5^. Cellular hypoxia is associated with the activation of a transcriptionally initiated response, mediated primarily by members of the hypoxia inducible factor (HIF) family (HIF-1α, HIF-2α and HIF-3α)^6^, and partially dependent on the accumulation of reactive oxygen species (ROS)^7^. Cellular oxygen sensitivity is achieved by two classes of enzymes, collectively termed HIF-hydroxylases (prolyl hydroxylases (PHD1–3)), also known as EGLN1–3 and factor inhibiting HIF (FIH). The regulation of HIF α-subunit protein stability is mainly achieved by oxygen-dependent posttranslational hydroxylation of conserved proline and asparagine residues. Under hypoxic conditions, these hydroxylation events are inhibited, leading to HIF-α protein accumulation and translocation to the nucleus. Subsequently, these HIF-α proteins will form a functional transcriptional complex that recognize the hypoxia response elements (HRE) elements of target genes^8^. HIF activation increases the expression of a number of genes, encoding proteins directed toward increasing tissue perfusion and oxygenation status, thus overcoming the initial hypoxic insult^9^. Since the 1990s^10,11^, previous research has shown that HIFs modulate the transcriptional response to hypoxia under both physiological and pathological conditions. This includes the expression of approximately 1000 genes that are broadly involved in the coordination of hypoxic adaptation; including those regulating metabolism, blood-vessel growth, cell division, inflammation and OxS response^12,13^.

However, the cellular response to hypoxia does not solely rely on members of the HIF family; other transcription factors (TF) are known to encode an extensive transcriptional program in response to a hypoxic stimulus^14^. Specifically, activation of nuclear factor kappa B (NF-κB) and nuclear factor (erythroid-derived 2)-like 2 (NRF2) are considered key regulating factors involved in the preservation of homeostasis in tissue and organs, attenuating oxidative damage, inflammation and modulating cellular adaptation. NF-κB and HIFs are considered the main players in inflammatory and innate immune responses with pro-or anti-inflammatory activity depending on which cell type is analyzed^15–19^. In particular, HIF-1α is widely expressed and is detected virtually in all innate and adaptive immune populations including macrophages, neutrophils, dendritic cells, and lymphocytes. Both NF-κB and HIF-1α could be considered master regulators of inflammatory gene expression involved in several relevant medical pathologies^20^. Conversely, NRF2 is the main TF involved in redox homeostasis and regulates a coordinated induction of anti-oxidant, and anti-inflammatory genes in response to OxS and inflammation^21–23^. It is clear that an extensive level of crosstalk between different TFs exists in response to a hypoxic insult, including a relationship between HIFs and NF-κB^19,20^, as well as NRF2 and NF-κB^24^. However, the timing and functional consequence of this concomitant induction of differing TFs during prolonged hypoxic exposure remains unclear.

Genomic studies have predominantly used cell culture and animal models to describe how exposure to hypoxia induces specific gene responses, as well as possible relationships between differing transcription factors^12,25–27^. However, the complex relationships between different TFs, also in relation to systemic effects of hypoxemia in humans *in vivo*, have not been fully elucidated yet. The decrease of partial pressure of oxygen with increasing altitude offers a unique experimental setting to investigate the cellular response to hypoxia *in vivo*^28^. In addition, white blood cells (WBC) have been recognized to be a viable *in vivo* model to investigate both continuous and intermittent hypoxia, inflammation and OxS in healthy humans^29,30^ and patients with cardiovascular diseases such as OSAS^31,32^.

We have hypothesized that acute hypobaric hypoxia (HH) exposure causes both increased inflammation and OxS, even in those subjects considered healthy, i.e. not exhibiting symptomology associated with altitude pathophysiology^1^. The proposition being that despite this collective initial increase in inflammatory and OxS response on immediate HH exposure, there exists a semi-concomitant activation of both anti-inflammatory and anti-OxS responses, differentially mediated by specific TFs depending on the duration of exposure ((i.e. sub-acute (≤24 h) or prolonged (72 h)). Specifically, we have investigated those molecular responses considered to monitor the expression pattern of the master cellular reprogramming transcription factors *HIF-1α*, *HIF-2α*, *NF-κB* (*p65* subunit) and *NRF2*, both before and during sub-acute to prolonged HH exposure in healthy participants. Furthermore, we have compared the expression pattern among those aforementioned TFs, as well as pro-inflammatory cytokines (*IL-1β* and *IL-6*), ROS production and OxS biomarkers (for DNA, lipid and protein peroxidation damage) over a 3-day exposure to HH equivalent to 3830 m. Due to a lack of clear consensus within the literature, we also chose to investigate the plasma levels of pro-inflammatory cytokines (IL-1β and IL-6). Previously described to increase under hypoxic conditions^33–35^, other publications have refuted this proposition, describing any hypoxia induced elevation of circulating pro-inflammatory cytokines as non-significant in healthy individuals^36,37^. Conversely, there is striking evidence that IL-6 may even be involved in hypoxia-induced lung inflammation and pulmonary vascular remodeling and then with the occurrence of specific high-altitude disease states^38^. Thus, further highlighting the necessity to investigate the temporal influence of HH exposure on IL-6 expression and IL-1β, which is not only proven to stimulate the synthesis of IL-6, but has also been identified generally as a critical mediator of tissue inflammation^39,40^. This study gives a unique insight into regulatory processes governing the early phase of hypoxic response in relation to inflammation and OxS in humans *in vivo*, as well as providing new knowledge specific to the molecular regulation of HIFs, and other master TFs, in a cellular population also implied in the pathogenesis of several cardiovascular diseases.

## Results

### Clinical parameters tested are indicative of physiological response during exposure to HH

A detailed overview of clinical parameters at baseline (BL), 24 h and 72 h is shown in Table 1. After direct ascent to high altitude, peripheral oxygen saturation (SpO_2_) showed a significant modification over time (ANOVA *P* < 0.001) with a reduction at 24 h (−11%, *P* < 0.001) and a progressive increase between 24 h and 72 h (+5%, *P* = 0.001). SpO_2_ did not return to the baseline values for any participant during stay at 3800 m. The heart rate (HR) increased from baseline values up until 24 h (+35%, *P* < 0.001), then HR decreased (−10%, *P* < 0.24), remaining higher compared to baseline (+21%, *P* < 0.006). Similarly, the breath rate (BR) enhanced within 24 h and after 72 h compared to baseline values (respectively +22%, *P* = 0.01 and +19%, *P* = 0.02). All the subjects included in data analysis had a value of Lake Louise Score (LLS) < 3 during three days of exposure to high-altitude hypoxia.

**Table 1:** The marked effects on human physiology of hypobaric hypoxia exposure over time is showed by the significative modification of blood capillary values SpO_2_ (peripheral capillary oxygen saturation) and clinical parameters such as Heart Rate (HR) and Breath Rate (BR).

	BL (n = 15)	24 h (n = 14)	72 h (n = 14)	Repeated measures ANOVA *P*	*P* after Bonferroni correction		
	Mean	Mean	Mean		24 h vs. BL	72 h vs. BL	24 h vs 72h
SpO_2_, %	98.93 ± 1.17	87.67 ± 3.48	91.71 ± 2.13	<0.001	<0.001	<0.001	0.001
HR, bpm	61.53 ± 7.99	83.07 ± 10.6	74.57 ± 13.69	<0.001	<0.001	0.01	0.24
BR*, rpm	13 ± 2.42	15.86 ± 3.01	15.44* ± 3.57	<0.001	0.01	0.02	0.87
LLS	0 ± 0	0.60 ± 0.74	0.36 ± 0.50	0.23	0.11	0.25	1

Only the BR seems to be less sensitive to hypoxia and its variations at specific time points are only marginally significant. Values are means ± SD. *BR was evaluated in 9 subjects.

### Exposure to HH results in differential expression of key TFs in human WBCs that is also time exposition related

**Figure 1:**
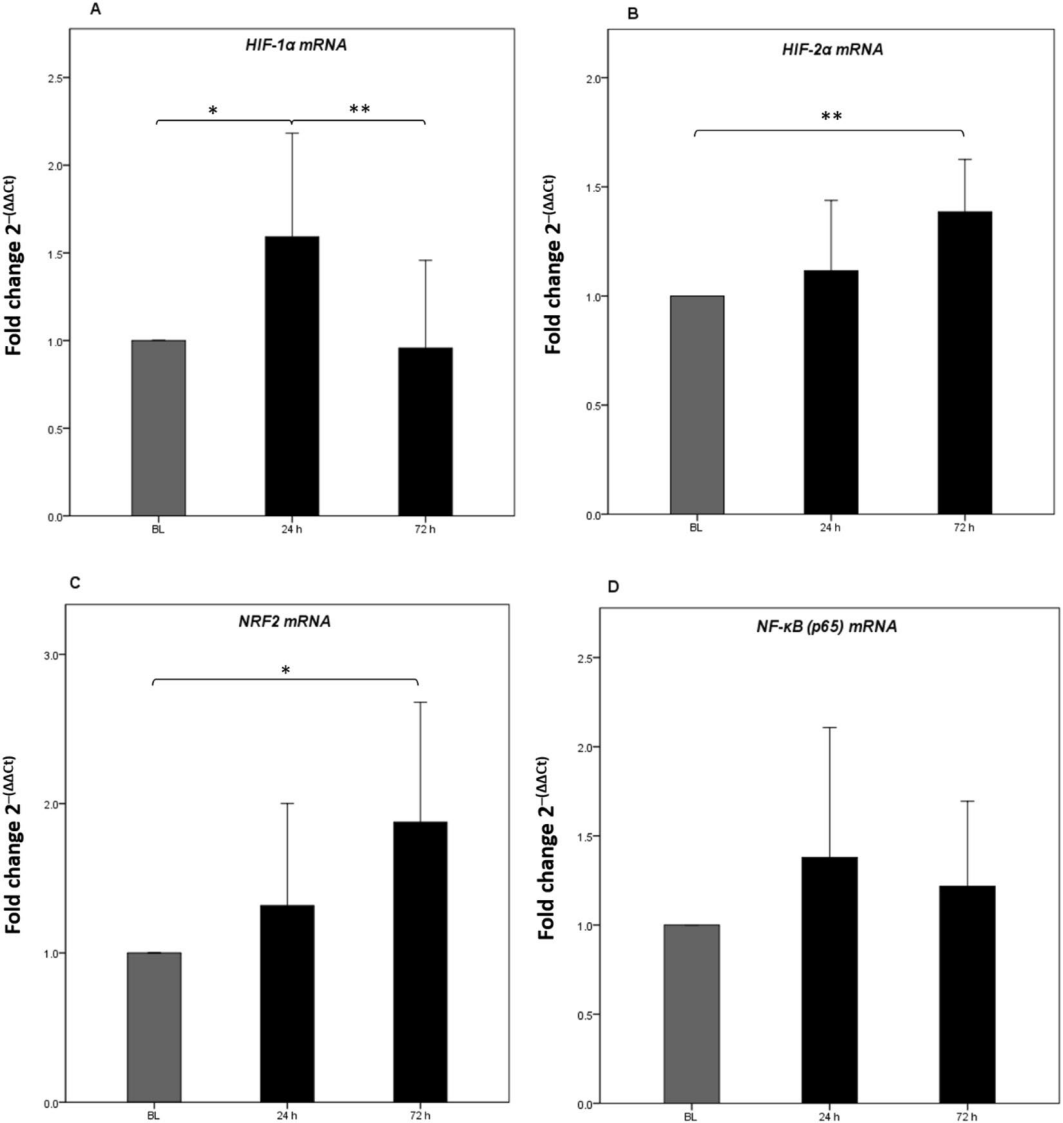
The relative quantification of mRNA levels for Transcription Factors *HIF-1α*, *HIF-2α*, *NRF2* and *NF-κB* (*p65*) in white blood cells reveal that a hypobaric hypoxia stimulus inducts different TF expression patterns that is time dependent. (**A**) *HIF-1α* shows a transcription peak within 24 h, while *HIF-2α* (**B**) and *NRF2* (**C**) show an expression peak after 72 h after the beginning of the stimulus. On the contrary, the *NF-κB* gene doesn’t show differences in its expression during the study (**D**). RQ was calculated as fold change using the 2^−(ΔΔCt)^ method. The results of mRNA are the average of the values assessed after three reaction tests. Values are mean ± SD. *P < 0.05 and **P < 0.001 after Bonferroni correction.

Gene expression of different TFs (*HIF-1α*, *HIF-2α*, *NRF2* and p65 subunit of *NF-κB*) were determined at baseline (262 m) and after 24 h and 72 h of high altitude exposure (3830 m). *HIF-1α* mRNA levels presented a parabolic relationship with time of exposure (72 h) to HH (ANOVA *P* = 0.001). Specifically, *HIF-1α* mRNA levels increased significantly after 24 h (+59%, *P* = 0.01) of hypoxic exposure, and subsequently returned to values comparable to baseline levels after 72 h (Supplementary Table S1 and Fig. 1A). *HIF-2α* mRNA levels consistently increased over time (ANOVA *P* = 0.001), reaching a peak after 72 h of exposure (+41%, *P* < 0.001) (Supplementary Table S1 and Fig. 1B). Similarly, the *NRF2* mRNA level increased constantly during hypoxic exposure (ANOVA *P* = 0.04), reaching a peak after 72 h (+87%, *P* < 0.001) (Supplementary Table S1 and Fig. 1C). Finally, in comparison to baseline values, *p65* mRNA levels were not significantly different after either 24 h or 72 h of high altitude exposure (ANOVA *P* = 0.71) (Supplementary Table S1 and Fig. 1D).

### Direct measurement of circulating pro-inflammatory cytokines during prolonged HH exposure indicate swift mediation of inflammatory state in HA responders

**Figure 2:**
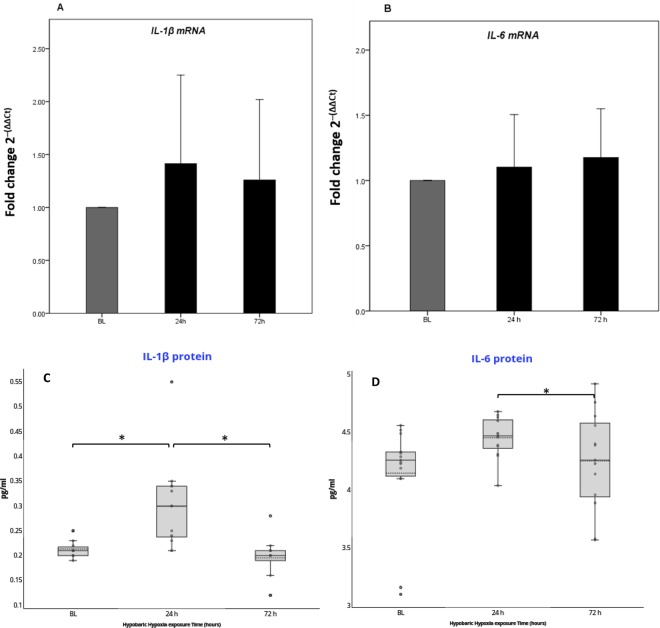
Relative quantification of mRNA levels and protein plasma levels for pro-inflammatory cytokines in white blood cells reveal a slight increase in inflammatory state during HH exposure. (**A**,**B**) *IL-1β* and *IL-6* mRNA quantification don’t reveal significant variation in mRNA production for both cytokines during hypoxia stimulus over time. The results of mRNA are the average of the values assessed after three reaction tests. Relative quantification of mRNA expression for pro-inflammatory cytokines was calculated as fold change using the 2^−(ΔΔCt)^ method. Values are mean ± SD. (**C**,**D**) Only IL-1β circulating protein showed a slight transient increase after 24 h of HH exposure. The absolute values for both circulating proteins are in the normal range for general population. The median (continuous band) and the mean (dotted band) are represented inside the boxes. *P < 0.05 after Bonferroni correction.

Gene expression analysis of pro-inflammatory markers (*IL-1β* and *IL-6*) were determined at baseline (262 m) and after 24 h and 72 h of high altitude exposure (3830 m). *IL-1β* and *IL-6* mRNA levels were not significantly modified during the 72 h period (ANOVA *P* = 0.65 and ANOVA *P* = 0.60 respectively) in WBCs (Supplementary Table S1 and Fig. 2A,B). On the contrary, IL-1β plasma levels significantly changed over time (ANOVA *P* = 0.001). Specifically, IL-1β plasma levels increased after 24 h (+25%, *P* < 0.02), and returned to baseline after 72 h (−2%, *P* = 0.77) (Supplementary Table S2 and Fig. 2C). IL-6 plasma levels did not significantly changed over time (ANOVA *P* = 0.07) (Supplementary Table S2 and Fig. 2D). However, we observed a significant reduction of IL-6 at 72 h of high altitude exposure, in comparison to values reported after 24 h (−5%, *P* = 0.04) (Supplementary Table S2 and Fig. 2D). Both plasma levels of IL-1β and IL-6 were considered in the normal range for the general population throughout the entire study period^41,42^. Within 24 h of exposure, 2 participants received a single dose of Paracetamol (500 mg). The adjustment of results for the use of paracetamol was performed, and no significant modifications were obtained (data not shown).

### Direct 24 h exposure to HH induces marked increases in ROS, TBARS, 8-isoPGFα, 8-OHdG, PC and concomitant reduction in TAC

To determine biomarkers of oxidative stress at baseline and during high altitude exposure, we measured the levels of ROS production, TBARS (Thiobarbituric acid reactive substances, markers of lipid peroxidation), 8-isoprostanes (8-isoPGF2α, lipid oxidation products and potential disease mediators), 8-hydroxy-2′ -deoxyguanosine (8-OHdG, products of DNA oxidation), PC concentrations (protein oxidative carbonylation) and TAC (total antioxidant capacity, the sum of aqueous and lipid soluble low-molecular weight antioxidants) for the whole cohort (Supplementary Table S3). OxS biomarkers levels at baseline (262 m) were in agreement with previous values estimated for a healthy population^43^. The basal level values for 8-isoPGF2α and 8-OHdG considered for statistical analysis were obtained from a matching group of the general population.

**Figure 3:**
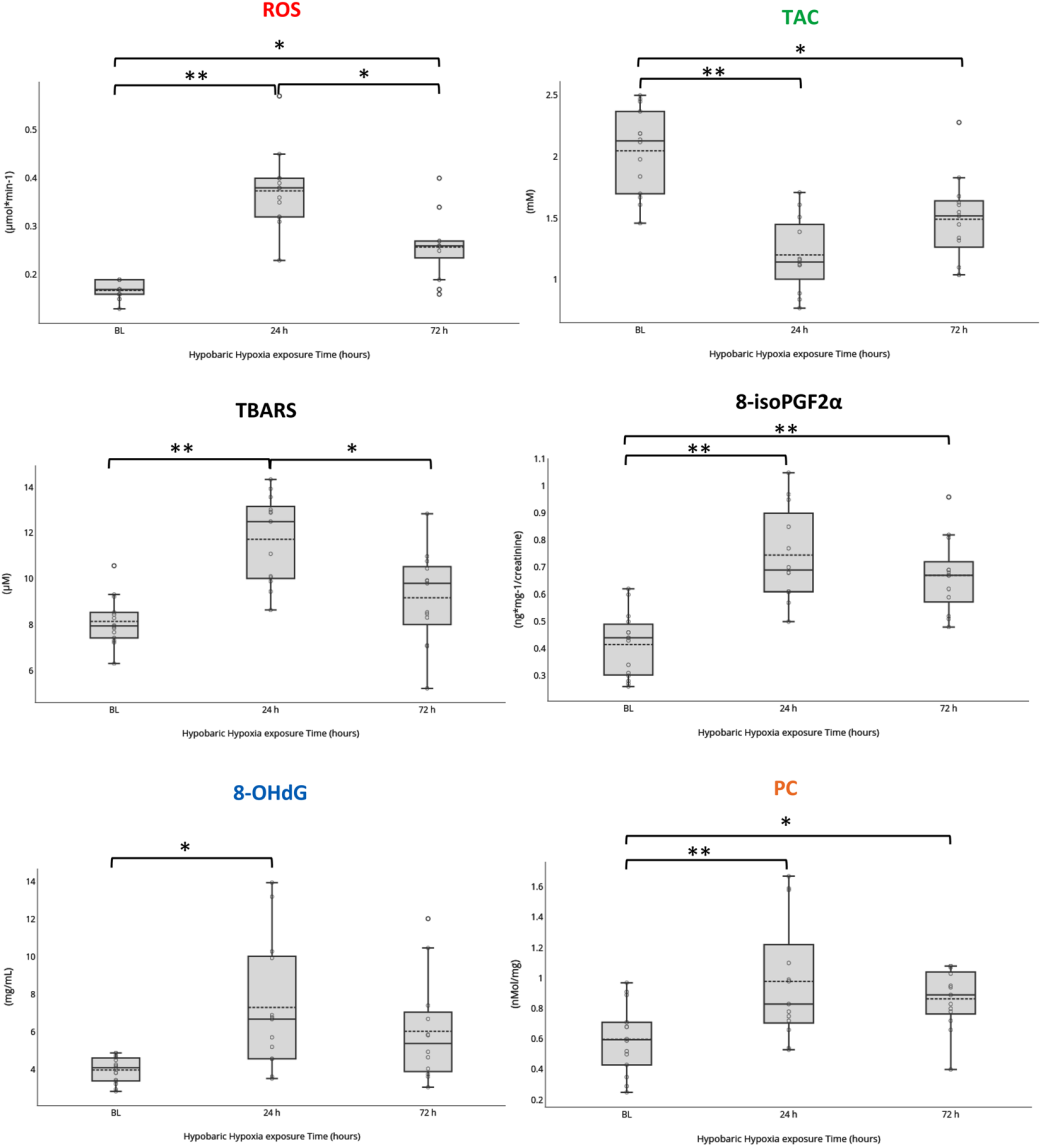
Box and Whisker plots show the marked effects of hypobaric hypoxia exposure (3830 m) on ROS production and relative biomarkers of oxidative damage. Direct 24 h exposure to HH induces marked increases in ROS, TBARS, 8-isoPGF α, 8-OHdG, PC and concomitant reduction in TAC. After 72 h of exposure to hypoxia there is a partial recover of the basal conditions with a slight improve of the TAC and a reduction of ROS and cellular damages amounts. The median (continuous band) and the mean (dotted band) are represented inside the boxes. *P < 0.05 and **P < 0.001 after Bonferroni correction.

Sub-acute HH exposure (24 h), led to changes in indices of OxS; namely: increased levels of ROS production as detected by EPR, TBARS, 8-isoPGF2α, 8-OHdG, PC, plus a decrease in total antioxidant capacity (TAC). Changes were detectable and reached a peak of +218% (ROS), +70% (TBARS), +54% (8-isoPGF2α) and +61% (PC) after 24 h (*P* < 0.001, *P* < 0.001, *P* = 0.001, *P* < 0.001 respectively) compared to baseline (Supplementary Table S3 and Fig. 3). Levels of 8-OHdG marker of DNA damages peaked after 24 h (+178%); the change was only slightly significant (*P* = 0.024) compared to BL. TAC values showed an exactly opposite pattern over time (ANOVA *P* < 0.001) with the lowest level of −60% after 24 h (*P* < 0.001) (Supplementary Table S3 and Fig. 3) and no restoring of the basal levels of antioxidant activity after the 72 h period of exposure (−25%, *P* = 0.001). Additionally, ROS production rate, TBARS, 8-isoPGF2α, 8-OHdG and PC concentrations significantly changed over time of exposure to HH within 72 h (ANOVA *P* < 0.001, *P* < 0.001, *P* = 0.002, *P* = 0.011, and *P* < 0.001 respectively) (Supplementary Table S3 and Fig. 3).

### Correlation among TFs gene expression, inflammatory and oxidative markers and clinical variables

We chose to perform a correlation analysis in order to investigate the tendency to show a coordinated expression among TFs, other studied genes (i.e. *IL-1β* and *IL-6*) related to OxS and inflammatory circulating markers (i.e. IL-1β and IL-6), over time. In particular, the correlation analysis was chosen to provide information pertaining to which biological processes were interconnected and which displayed interdependence. The main aim was to identify which potential interactions may be of mechanistic importance in the early phase of human response to hypobaric hypoxic exposure.

### Clinical variables showed a strong correlation with ROS and TAC but not with TFs and inflammation markers

SpO_2_ showed strong correlations with both ROS production (ANOVA r = −0.76, *P* < 0.001) and TAC (ANOVA r = +0.82, *P* < 0.001) over time (Table 2). TAC and ROS were also correlated (ANOVA r = −0.66, *P* < 0.001) (Table 2 and Supplementary Fig. S1).

**Table 2:** SpO_2_ showed a strong correlation with ROS and TAC but a weak correlation with TFs and inflammation markers.

correlation with	Clinic.				Transcritpion Factors						Oxidative stress				Inflammatory state							
	SpO_2_		*HIF-1α*		*HIF-2α*		*NF-κB*		*NRF2*		ROS		TAC		*IL-1β*		*IL-6*		IL-1β protein		IL-6 protein	
	r	P	r	P	r	P	r	P	r	P	r	P	r	P	r	P	r	P	r	P	r	P
SpO_2_	—																					
*HIF-1α*	−0.41	0.03	—																			
*HIF-2α*	−0.33	0.09	−0.19	0.34	—																	
*NF-κB*	−0.47	0.01	0.31	0.12	0.14	0.49	—															
*NRF2*	−0.42	0.03	−0.29	0.15	0.58	0.002	0.30	0.13	—													
ROS	−0.76	< 0.001	0.62	< 0.001	0.15	0.46	0.46	0.02	0.15	0.45	—											
TAC	0.82	< 0.001	−0.37	0.07	−0.24	0.25	−0.59	0.002	−0.43	0.03	−0.66	< 0.001	—									
*IL-1β*	−0.37	0.06	0.08	0.69	0.34	0.08	0.29	0.14	0.30	0.12	0.36	0.07	−0.32	0.12	—							
*IL-6*	−0.10	0.63	0.10	0.61	0.46	0.02	0.16	0.43	0.15	0.45	0.32	0.11	−0.03	0.89	0.40	0.04	—					
IL-1β protein	−0.46	0.02	0.38	0.06	−0.13	0.52	0.49	0.01	−0.02	0.92	0.47	0.02	−0.35	0.08	0.32	0.12	−0.06	0.76	—			
IL-6 protein	−0.44	0.02	0.26	0.21	0.21	0.32	0.47	0.02	0.17	0.43	0.34	0.09	−0.47	0.02	0.18	0.40	−0.04	0.84	0.37	0.05	—	

Additionally, the TFs showed a differential correlation with both OxS markers and inflammatory variables. In particular, *HIF-1α* mRNA was correlated with ROS over time, as well as *HIF-2α* with *NRF2*. All P-values were two sided, and differences were considered significant at *P* < 0.01; 0.01 < *P* < 0.05 were considered only marginally significant.

SpO_2_ showed a negative but marginally significant correlation with three out of four TFs investigated (*HIF-1α*, *NF-κB* and *NRF2*) (ANOVA r = −0.41, *P* = 0.03; ANOVA r = −0.47, *P* = 0.01 and ANOVA r = −0.42, *P* = 0.03 respectively).

SpO_2_ showed a marginally significant correlation with IL-1β and IL-6 protein levels in plasma (ANOVA r = −0.46, *P* = 0.02; ANOVA r = −0.44, *P* = 0.02). IL-1β and IL-6 protein concentrations showed a marginal significant correlation with ROS (ANOVA r = +0.47, *P* = 0.02 and r = +0.34, *P* = 0.09 respectively) and TAC (ANOVA r = −0.35, *P* = 0.08 and r = −0.47, *P* = 0.02 respectively) over time. Only a marginal significant correlation was identified between *IL-1β* mRNA and *IL-6* mRNA (ANOVA r = +0.40, *P* = 0.04) levels in WBCs. On the contrary no correlations were observed between the mRNA of the two investigated interleukins and their protein concentrations in plasma (ANOVA r = +0.32, *P* = 0.12 and ANOVA r = −0.04, *P* = 0.84 respectively). Finally, IL-6 protein showed a marginally negative correlation with TAC (ANOVA r = −0.47, *P* = 0.02).

### Transcription factors showed a differential correlation with each other, OxS markers and inflammatory variables

*HIF-1α* mRNA was not correlated with *HIF-2α* (ANOVA r = −0.19, *P* = 0.34) and *NRF2* mRNA (ANOVA r = −0.29, *P* = 0.15) whereas *HIF-2α* mRNA levels were positively correlated with *NRF2* gene expression over time (ANOVA r = +0.58, *P* = 0.002) (Table 2 and Supplementary Fig. S1).

*HIF1α* mRNA was positively correlated with ROS overtime (ANOVA r = +0.62, *P* < 0.001) (Table 2). *HIF-2α* mRNA levels showed a marginally significant correlation with the *IL-6* mRNA levels over time (ANOVA r = +0.46, *P* = 0.02) (Table 2 and Supplementary Fig. S1).

*NRF2* mRNA level was negatively correlated with TAC (ANOVA r = −0.43, *P* = 0.03). Significant correlations were identified involving *NF-κB* mRNA with TAC and ROS (ANOVA r = −0.59, *P* = 0.002 and ANOVA r = +0.46, *P* = 0.02). Only marginal positive correlations were identified involving *NF-κB* mRNA and plasma interleukins IL-1β and IL-6 overtime (ANOVA r = +0.49, *P* = 0.01 and ANOVA r = +0.47, *P* = 0.02 respectively).

## Discussion

These data describe, for the first time *in vivo* in human WBCs, the differential regulation of specific transcription factors in response to both sub-acute (≤24 h) and prolonged (72 h) sedentary HH exposure in healthy participants post direct ascent to 3830 m. Specifically, these data highlight: i) the individual contribution of *HIF-1α* and other master TFs (*HIF-2α*, *p65* and *NRF2*) involved in the mediation of an HH response; ii) the concurrent transcription pattern of these TFs, and iii) the relationship demonstrated with established inflammatory and OxS pathways over time. During this study confounding factors (e.g., physical exertion, changes in HH level, cardiovascular as well as other chronic diseases, and diet) were minimised. Therefore, we infer that our results reflect a ‘clean’ model for the interrogation of the early phase of WBC response to both a sub-acute and prolonged HH stimulus. This integrated analysis shows that HIF-1α is a lead TF during the sub-acute hypoxic response, preventing an excessive pro-inflammatory response, as well as contrasting the proposed collective repression of antioxidant activity under HH conditions. In addition, we have showed that the prolongation of HH stress led to a switch in predominant TF induction. In particular, after 24 h a significant increase in both the transcription of the *HIF-2α* isoform and *NRF2* is evident. This relative, sustained increase is concomitant with a decline in *HIF-1α* transcription, thus suggesting the presence of a synchronized mechanism that led to an effective molecular anti-inflammatory “brake” in response to acute HH exposure. This anti-inflammatory activity is in direct contrast to the previous proposition for collective systemic inflammation.

*HIF-1α* mRNA levels demonstrated a transient increase within 72 h of HH exposure, reaching a peak after 24 h and returning to a basal level within 72h^43,44^. These *in vivo* results, in humans, demonstrate a preferential involvement of the *HIF-1α* gene transcription during the early phase of HH stress response. Equally, this parabolic response supports the hypothesis that HIF-1α steady state basal protein levels, plus post-transcriptional and post-translational regulations alone are not effective in response to such a direct HH exposure (helicopter ascent to 3830 m)^43,45^. Our results confirm that prolonged HH exposure appears to favor the transcription of the *HIF-2α* gene, with a progressive increase in expression after 24 h of exposure^14^. Which potentially indicates a definitive switch in HIFs regulation, from the initial cellular defense to a sudden hypoxic stressor, to that of a sustained cellular response to a prolonged hypoxic stimulus. Overall, our *in vivo* results in WBCs seem consistent with the HIF switch model observed in previous *in vitro* studies, both in vascular and bone development, and cancer stem cells^25,46^. Differences in downstream target genes^12,25^ could partially explain why HIF-1α drives the initial response to HH. HIF-1α leading the homeostatic defense with apoptotic and glycolytic enzyme target genes, whereas HIF-2α potentiating the physiological response during prolonged HH exposure via angiogenic and invasion target genes as showed also by recent studies of evolutionary genomics in populations living permanently at high-altitude^47^. Moreover, we demonstrated that *NRF2* mRNA significantly increased in expression over time, reaching a peak after 72 h, in parallel to the expression of *HIF-2α*.

Previous experimental studies have suggested that NF-κB, and NRF2 are pleiotropic TFs that have been implicated in many cellular processes, including inflammation and OxS responses to hypoxic exposure^21,23,48^. The NF-κB subunits p50 and p65 are, in fact, activated by hypoxia and mediate the induction of *HIF-1α* mRNA via a transcriptional mechanism by binding to the *HIF-1α* promoter, thus suggesting a specific role of NF-κB in the maintenance of basal *HIF-1α* mRNA levels^43^. However, in this *in vivo* study the *p65* mRNA levels were not significantly upregulated, neither were they correlated with *HIF-1α* gene expression or the expression of other TFs. In addition, only a slightly significant positive correlation with interleukin protein levels in plasma and a negative correlation with TAC response is observed as expected throughout 72 h of HH exposure. The lack of NF-κB subunit activation is reported in contrast with previous studies using hepatocellular carcinoma culture, HeLa cells and *ex vivo* cardiac and pulmonary tissues harvested from hypoxic mice, where an increased transcription of *NF-κB* isoforms was repeatedly demonstrated with the application of a hypoxic stimulus^49,50^. These findings may be explained by the fact that in those previous studies *NF-κB* isoform transcription and/or activation was heavily dependent on cellular type tested and specific experimental model. Currently, we cannot exclude the possibility that *p65* expression could be significantly enhanced in specific white cell types, an effect which could have been masked in our study due to the consideration of a global WBC population. On the other hand, our findings are in agreement with the idea that HIF-1α could restrain the NF-κB response and thus prevent excessive and damaging pro-inflammatory responses^16^.

Several papers have previously reported that HH, similarly to cardiovascular diseases and other disease states, can lead to an increase in the inflammatory state^33,34,51^, accompanied by a significant increase in circulating pro-inflammatory cytokines. In correspondence, our results have shown a marginally negative correlation between SpO_2_, circulating IL-1β and IL-6 plasma levels. In the meanwhile, a marginally positive correlation among the two circulating cytokines was observed as expected by previously reported information^39^. IL-1β plasma levels transiently increased over 72 h, however the absolute value remained in the normal range expected for healthy individuals^41^. In addition, IL-6 plasma levels were also considered in the normal range for healthy individuals throughout our 72 h HH protocol^45^. In contrast with protein results at a cellular level, in WBCs, there was no significant increase of *IL-1β* mRNA and *IL-6* mRNA and only a marginal significant positive correlation was observed between them over time. Therefore, no significant correlation was observed among investigated interleukin mRNA expression and their respective proteins, this is in agreement with the idea that circulating interleukins are released by different tissues and not exclusively by WBCs. Altogether, these data suggest that despite the expected initial increase in inflammation on rapid, direct ascent to HH, any major discrepancy in circulating pro-inflammatory cytokines may be quickly and progressively counteracted by an effective anti-inflammatory action. This activity is probably mediated primarily by HIF-1α^19,23,24^ and later surpassed by a concomitant upregulation of *NRF2*^52^. Our *in vivo* results are in agreement with other papers that did not show increased plasma levels of pro-inflammatory cytokines in healthy and acute mountain sickness prone subjects^36,37,53^. Within this topic, with reference to similar studies, comparative analysis is challenging to complete and discrepancies are likely due to specific differences in study design, cytokine dosage protocol^35^ and statistical analysis.

HH exposure leads intrinsically to an increased OxS in healthy humans, as well as in animal models^1,53,54^. Our results confirm that OxS is strongly increased in particular within 24 h of exposure to HH, as shown up by the increased oxidative damage of biomolecules such as lipids, proteins and DNA. Moreover, the positive correlation among SpO_2_ and TAC, plus the contemporary negative correlations between SpO_2_ and TAC with ROS, both support the idea of an initial delay of effective antioxidant defense, followed by delayed ROS species scavenging^1,3,55,56^. Our results are in agreement with the idea that the pathways by which ROS could contribute to cell signaling responses to hypoxia involves the HIF family and NRF2. NRF2 is the guardian of redox homeostasis and regulates a coordinated induction of a battery of cytoprotective, anti-oxidant, and anti-inflammatory genes in response to OxS and inflammation^21–23^. *NRF2* mRNA levels in WBC increased constantly over time showing a positive correlation with *HIF-2α* mRNA levels (ANOVA) and a positive correlation with TAC (ANOVA). Our *in vivo* results suggest that the HH-induced ROS formation leads to an effective HIF-1α and NRF2 activity, only after reaching a critical threshold that seems defined by both level of hypoxia and duration of exposure^57,58^. Indeed, these results demonstrate that within 72 h the ascent protocol used in this study was able to identify differing temporal regulation of pertinent cellular factors involved in hypoxic response. This includes, those factors necessary for initial hypoxic cellular defense (HIF-1α) and those considered more active in the regulation of prolonged cellular response (HIF-2 α and NRF2). However, the duration of the exposure was not long enough to characterise the level of expression in all selected factors, and therefore cannot be used to infer cellular regulation during a complete and effective acclimatisation.

The positive correlation between *HIF-1α* and ROS are in agreement with the idea that HIF-1α is stabilized in hypoxia via the hydroxylation of different prolyl group by proline hydroxylases, this activity has been shown to be sensitive to inhibition by ROS^59^. Finally, we consider the marginally significant positive correlation observed among the IL-1β protein with ROS, and the similarly marginal significant negative correlation of IL-6 protein with TAC, as potential inference for existing interdependent and interconnected processes among inflammation and OxS during hypoxia response in humans^60,61^.

The current study has several caveats. Firstly, TF protein levels and activities were not measured due to logistical and ethical limitations related to the study design (e.g. barometric pressure-decreased PAXgene blood RNA tubes sampling; repeated samples in humans in a field study at altitude). Nevertheless, transcriptional regulation is an important pre-cursor of functional TFs. Secondly, a larger sample size, with additional sampling time-points both during and after hypoxic exposure, including information related to individual “reoxygenation profiles” after return to sea level, would have improved our study model. In future studies, inclusion of information relating to the molecular impact of re-oxygenation post prolonged hypoxic exposure, could be pertinent when considering the mechanistic aspect of specific hypoxia related pathologies (i.e. ischemia-reperfusion). Equally, in this study it would have been helpful to determine novel TF-oriented markers, such as HIF-3α, p50 (NF-κB family member), and TLRF2. Nevertheless, the aim of the study was to try to describe for the first time, in humans *in vivo*, the temporal mediation of possible master TFs involved in the hypoxemic, inflammatory and OxS pathways in response to both sub-acute and prolonged HH exposure. Finally, it remains unknown as to when TFs like *NRF2* and *HIF-2α* are, in fact, repressed; thus establishing a new steady state and avoiding deleterious effects of an excessive prolonged activation of target genes involved in angiogenesis and red blood cell formation. Further microRNAs and epigenetic studies are required to better elucidate the detailed aspects of the intricate and complex crosstalk between those TFs and pathways reportedly activated or repressed during hypoxic response^20,24^.

## Conclusions

**Figure 4:**
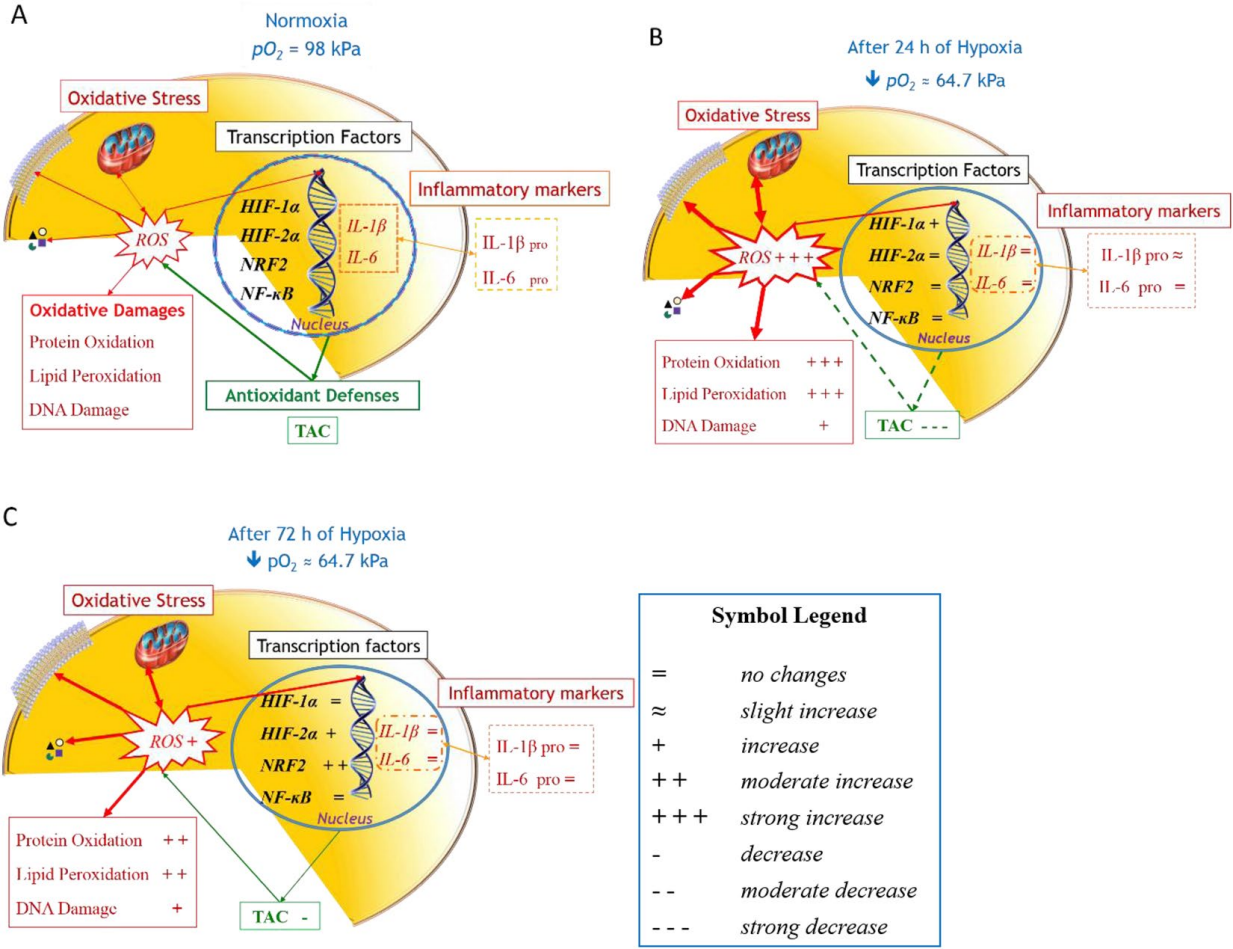
HH response in humans is governed by both spatial and temporal mediation of the OxS and inflammatory pathways via a series of micro and macro events. This schematic overview highlights qualitatively how pertinent relationships between crucial molecular transcription factors and both oxidative and inflammatory pathways involved in the cellular hypoxic response are mediated over time. The +, =, and **−** symbols refers to qualitative changes of variables during hypoxia exposure in comparison with baseline. (**A**) During normoxic conditions a well-balanced level of ROS cytokines and TFs mRNA turnover are present in the cells. (**B**) After 24 h of HH exposure *HIF-1α* mRNA transcription is enhanced but the level of OxS is also high, plus the existing antioxidant defenses fielded by the cell seem ineffective, results demonstrating a delayed induction of *NRF2*, in contrast to the immediate deleterious impact of ROS induction on key cellular biomolecules such as lipids, proteins and DNA. On the contrary, the slight increase in circulating IL-1β demonstrates an early attempt by the cell to mitigate the inflammation response. (**C**) After 72 h of exposure the situation is dramatically changed, showing a marked reduction of ROS production and a consequent reduction of damages on biomolecules, probably counteracted by the scavenging activity performed by antioxidant enzymes. Conversely, no significant modifications were shown by either inflammatory markers or *NF-κB* after 72 hours of constant, passive hypobaric hypoxic exposure.

We have identified *in vivo* differential temporal regulation of molecular aspects of HIFs and other master TFs in healthy humans in direct response to HH-induced inflammation and OxS (Fig. 4). Our integrated analysis of TF-gene expression suggests that HIF-1α is a lead TF during the early phase of direct HH exposure, preventing an excessive pro-inflammatory response^16^ and contrasting the repression of antioxidant activity on initial exposure to high altitude (Fig. 4A,B). Correspondingly, we have showed that prolonged HH exposure results in the sequential induction of differentially mediated anti-inflammatory activity; most likely probably due to the contemporary repression of the pro-inflammatory role of NF-κB (Fig. 4C). Specifically, the activation of HIF-1α, HIF-2α and NRF2 appear to act in concert, in an attempt to establish an effective molecular anti-inflammatory “brake” in response to the evident systemic inflammation induced by prolonged HH. The *in vivo* values of ROS and OxS-damage biomarkers confirmed a prolonged transient increase, due to a delay in the activation (and effectiveness) of the NRF2-mediated antioxidant defenses. Results from humans exposed to an acute and prolonged hypoxic stimulus offer a unique means to evaluate *in vivo* the time-related response/counteraction measures to inflammation and OxS. Our results have demonstrated that the extensive transcriptional output of HIF in response to hypoxia is highly synchronized and integrated with additional signaling pathways; involving nutrient sensing, protein synthesis and other fundamental processes. All the aforementioned TFs and mechanisms have a clinical relevance and are considered potential therapeutic targets for cardiovascular and other diseases that are associated with OxS and inflammation^3,13,62,63^. The failure of supplementation with exogenous antioxidants vitamins for prevention or treatment of cardiovascular disease reinforced the idea of alternative approaches to counteract the deleterious effects of an excessive ROS production^64,65^. In particular, the therapeutic potential of phytochemical and synthetic compounds for *NRF2* activation in attenuating oxidative damage associated in neurodegenerative diseases, cancer and cardiovascular diseases has been previously shown and discussed^63–65^. The beneficial role of HIF-1α activation is shown in the revascularisation and collateral circulation in ischemic brains in rodents^66^. On the contrary, it has been demonstrated that HIF-1α plays a role in the progression of atherosclerosis suggesting that the repression of its activity could lead to beneficial outcome for patients^62^.

To date, the hypoxic preconditioning is used as a therapeutic strategy to mitigate injuries in the cardiovascular system, stimulating and/or repressing factors that are important players in anti OxS and inflammation. Our *in vivo* study provides new details on the complexity of the cross-talk and temporal regulation of molecular mechanisms and factors (HIF-1α, HIF-2α and NRF2) involved in cellular response to ROS and inflammation during a hypoxic stimulus. Our results could influence the design of new clinical trials inclusive of hypoxic protocols or hypoxia mimetics for therapeutic use, where definition of TF induction with specific hypoxic doses remains to be defined. A separate focus could include testing the role of HIF-2α as a therapeutic target^62,67^, the aim to further distinguish protocols intended to study acute and/or chronic hypoxia pathologies.

## Methods

### Study design and participants

**Figure 5:**
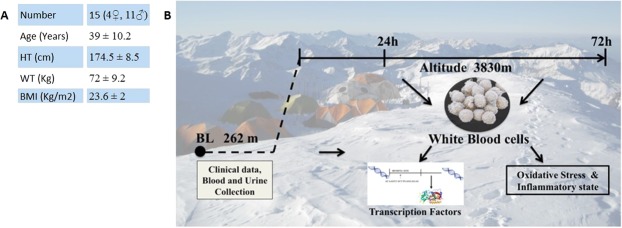
(**A**) Table of human volunteer characteristics, including age, height (HT), weight (WT), body mass index (BMI) (**B**). Outline of study design, and ascent profile.

From the initial sample, including 19 healthy participants of European ancestry, 15 completed the experimental period. Participant’s characteristics are reported in Fig. 5A. Prior to the study, none of the participants were treated with either acetazolamide, non-steroidal, or steroidal anti-inflammatory agents. Exclusion criteria during selection were: age <18 years and habitual intake of antioxidant or anti-inflammatory substances at baseline visit; acute illness (infectious, cardiovascular, cerebrovascular or respiratory), and any prior acute high-altitude illness. One participant was undergoing hypertension therapy and one of the participants was reportedly a smoker. During the study, only non-steroidal anti-inflammatory agents were permitted after consultation with the study’s physician. Moreover, the Lake Louise Score (LLS)^68^ was assessed to evaluate the health status of participants. Subjects with a LLS ≥ 3 were excluded from the study.

Written informed consent was obtained from all participants. The study was approved by the Ethics Committee of Bolzano General Hospital (number 0073450-BZ), and conformed with the Declaration of Helsinki (2008). The study is registered at www.clinicaltrials.gov (registration number NCT01794130).

Two weeks prior to experimental ascent, participants underwent baseline medical examinations at the Institute of Mountain Emergency Medicine, Eurac Research, Bolzano, Italy (262 m). These examinations were subsequently repeated in a medical tent located near Mt. Ortles summit, Italy, both 24 h and 72 h post direct ascent by helicopter to 3830 m (Fig. 5B). Participants remained in tented accommodation at 3830 m for a total of three days, during which they were instructed to avoid any unnecessary physical exertion.

### Clinical assessment and high altitude health conditions

Medical examinations included the assessment of: oxygen saturation (SpO_2_; Handheld Pulse Oximeter NBP-40, Nellcor Puritan Bennet Inc., Pleasanton, CA, USA); heart rate (HR) and breathing rate (BR). SpO_2_ and HR were both measured at rest in triplicate using the same device, care was taken to ensure signal stabilisation and that participants had warm hands on examination. Both were reported as an average of three consecutive measurements.

Throughout the study, the Lake Louise Score (LLS) and a number of alternative signs and symptoms were assessed to evaluate the health status of participants including dyspnea, cough, fatigue, cyanosis, and rales.

During the 3 day study period, those individuals who presented at least once with a LLS ≥ 3 (including a headache score ≥ 1) were considered bad responders and excluded.

### Blood and urine samples collection

Venous blood samples were drawn from the antecubital vein, collected in heparinized tubes (Becton Dickinson Company, UK) centrifuged and separated. Plasma was stored in aliquots at −80 °C until analysis. In addition, urine samples were collected and stored at −80 °C. Moreover, for each participant 5 mL of venous blood were collected into two PAXgene® blood RNA tubes (i.e. 2 × 2.5 mL) (QIAGEN, Mississauga, Ontario) and stored in multiple aliquots at −80 °C until assayed for gene expression quantification.

### Candidate genes expression levels

#### Blood processing, RNA isolation, mRNA determination

Total RNA from whole blood was extracted using the PAXgene® Blood RNA Kit (PreAnalytiX), according to the manufacturer’s instructions, to evaluate the white blood cell expression. RNA quantity and quality were determined by the ratio from the absorbance readings at 260 nm and 280 nm with a NanoDroP 2000C (Thermo Scientific, USA).

For gene expression, cDNA was synthesized with 200 ng of RNA extracted using iScript cDNA synthesis kit (Bio-Rad, Hercules, CA) according to the manufacturer’s instructions.

### Genes expression

Quantitative real-time polymerase chain reaction assay was performed in a Bio-Rad CFX96 Real Time PCR detection system^69^. All the reactions were performed in 96-well plates, in triplicate. A negative control containing all reagents, but no cDNA template was included in all runs.

The PCR reaction was performed in a 25 μL final reaction volume, containing 200nmol of each primer and SsoFast EVAGreen SuperMix (Bio-Rad, USA). Primers were designed from sequences derived from the GenBank database using Primer 3 (Whitehead Institute, Massachusetts, USA) and Operon’s Oligo software (Operon, California, USA). Primers were purchased from Eurofins MWG (Ebersberg, Germany). The specific primers were (Eurofins): *HIF-1α*, Forward ACAAGCCACCTGAGGAGAG and reverse AAGTGGCAACTGATGAGCAAGC; *HIF-2α*, Forward TGTATGGTCAGCTCAGCCC and reverse GCTCCACCTGTGTAAGTCCC; *IL-6*, Forward TTAGAGTCTCAACCCCCAATAAAT and reverse TACATGTCTCCTTTCTCAGGGC; *β-actin*, Forward GAGCTACGAGCTGCCTGAC and reverse GGATGCCACAGGACTCCA. Validation of specificity of qPCR assay was performed by melting-curve analysis and agarose gel analysis. *NF-κB* (p65) (Hs00153294), *IL-1β* (Hs00174097), *NRF2* (Hs00975961) gene expression were measured using Taqman primers (Applied Biosystems, USA). All procedures were performed according to the manufacturer protocols. *β-Actin* was used as reference gene. For each target gene, a calibration curve was generated with threshold cycle (Ct) values from serial dilutions of cDNA (from 10^6^ to 10 copies/reaction) to determine reaction efficiencies, linearity, detection and quantification limits. Data analyses were performed with the Bio-Rad CFX Manager. The comparative cycle threshold method (ΔΔCt), which compares the difference between groups in cycle threshold values, was used to obtain the relative fold change of gene expression.

### Inflammatory markers assay

Interleukin 1β (IL-1β) and Interleukin 6 (IL-6) plasma levels were determined by ELISA assay kit (ThermoFisher Scientific, CA, USA), based on the double-antibody “sandwich” technique according to the manufacturer’s instruction. The assay sensitivity was <0.92 pg.mL^−1^ and <0.06 pg.mL^−1^ for IL-6 and IL-1β respectively.

### ROS detection and Oxidative damage assessment

#### Plasma ROS measurements

An X-band EPR instrument (E-scan-Bruker BioSpin, GmbH, MA) was used to determine ROS production. The instrument is designed to function with very low concentrations of paramagnetic species in small (50 μL) samples. For each recruited participant, the ROS production rate was determined by means of a recently implemented EPR method^1,70^.

#### Plasma Total Antioxidant Capacity (TAC) determination

TAC was measured via enzymatic kit (Cayman Chemical, Ann Arbor, MI, USA), based on the ability of antioxidants present in the plasma to inhibit the oxidation of 2,2-azinobis (3-ethylbenzithiazoline) sulfonic acid (ABTS) to the radical cation ABTS+ by a peroxidase. The amount of the produced ABTS+ was assessed by measuring the absorbance signals at 750 nm. TAC was evaluated by a Trolox (6-hydroxy-2,5,7,8-tetramethylchroman-2-carboxylic acid) standard curve and was expressed as trolox-equivalent antioxidant capacity concentration (mM).

#### Protein Carbonyls (PC) determination

The accumulation of oxidized proteins was measured by content of reactive carbonyls. A protein carbonyls assay kit (Cayman Chemical, Ann Arbor, MI, USA) was used to evaluate colorimetrically oxidized proteins at 370 nm. Values obtained were normalised to the total protein concentration in the final pellet (absorbance reading at 280 nm), to consider protein loss during the washing steps, as suggested in the kit’s user manual.

#### Plasma Thiobarbituric acid-reactive substances (TBARS) determination

The measurement of TBARS was determined by TBARS assay kit (Cayman Chemical, Ann Arbor, MI, USA), which allows a rapid photometric detection of the thiobarbituric acid malondialdehyde (TBAMDA) adduct at 532 nm.

#### Urine 8-isoprostane (8-isoPGF2α) determination

EIA assay was used for the determination of 8-iso PGF2α (Cayman Chemical, Ann Arbor, MI, USA). Samples and standards were read at a wavelength of 412 nm. The results were normalized by urine creatinine values.

#### Urine 8-OH-2-deoxyguanosine (8-OH-dG) determination

8-OH-dG was measured by immunoassay EIA kit (Cayman Chemical, Ann Arbor, MI, USA) as a biomarker for oxidative damage. Samples and standards were read at a wavelength of 412 nm. The results were normalized by urine creatinine values.

Creatinine: Urinary concentrations of 8-iso PGF2α and 8-OH-dG, as any urinary marker, vary considerably; therefore, the urinary parameters are usually standardised based on the amount of creatinine excreted in the urine when the collection of the 24 h urine is not possible. Indeed, in the absence of renal disease, the excretion rate of creatinine in an individual is relatively constant. Thus, urinary creatinine levels may be used as an index of standardisation. A creatinine assay kit (Cayman Chemical, Ann Arbor, MI, USA) was used to measure creatinine levels in urine samples.

### Statistical analysis

The effect of HH exposure on the different clinical parameters and gene expression over time was evaluated by analysis of variance (ANOVA) for repeated measures of ΔCt for gene mRNA levels; comparison of two time-points was carried out by means of paired samples *t*-test and Bonferroni correction was applied. A correlation coefficient for repeated observations was used to detect if changes over time of two parameters were correlated. The relative quantification of genes was determined using the 2^−ΔΔCt^ method.

Data were analyzed using SPSS 23.0 statistical software package (IBM Corp., Armonk, NY, USA). Variables such as sex, altitude of residence, and smoker status were considered in the statistical analysis, but no confounding influence was found on results (data not shown). All *P*-values were two sided, and differences were considered significant at *P* < 0.05; for correlation analysis *P* < 0.01 was considered significant and *P* < 0.05 marginally significant. Plotly software (Plotly Technologies Inc., Montréal, Québec, CA) was used to draw Box and Whisker plots (https://plot.ly/).

## Supplementary information

Supplementary Info

## Data Availability

The authors confirm that the data supporting the findings of this study are available within the article and its supplementary materials.
