# Supplementary material for: Transcription Factors Regulation in Human Peripheral White Blood Cells during Hypobaric Hypoxia Exposure: an in-vivo experimental study
Source: Sci Rep. 2019 Jul 9;9:9901. doi: 10.1038/s41598-019-46391-6 (PMC6617471; doi:10.1038/s41598-019-46391-6)
Supplement: Supplementary file 1 — Supplementary Info [file 41598_2019_46391_MOESM1_ESM.doc]

**Supplementary Information**

**Transcription Factors Regulation in Human Peripheral White Blood Cells during Hypobaric Hypoxia Exposure: an *in-vivo* experimental study**

**By**

*Sandro Malacrida, Alessandra Giannella, Giulio Ceolotto, Carlo Reggiani, Alessandra Vezzoli, Simona Mrakic-Sposta, Sarah Moretti, Rachel Turner, Marika Falla, Hermann Brugger, Giacomo Strapazzon.

***Correspondence to:** Sandro Malacrida, Institute of Mountain Emergency Medicine, European Academy of Bolzano; Via Drususallee 1; 39100 Bolzano, Italy. Email: [sandro.malacrida@eurac.edu](mailto:sandro.malacrida@eurac.edu)

**Supplementary Tables and Figures**

**Supplementary Table S1**. Mean mRNA expression levels of transcription factors and proinflammatory cytokines in blood reveal that a hypobaric hypoxia stimulus inducts a peak in gene expression at different times. *HIF-1α* maximum mRNA level is measured within 24 h of exposure whereas *HIF-2α* and *NRF2* show a peak level after 72 h of exposure. Values are means of ΔCt ± SD.

**Supplementary Table S2**. Mean protein expression levels of proinflammatory cytokines in plasma reveal a slight change in IL-1β protein levels: suggesting a moderate inflammatory stimulus within 24 h. On the contrary the IL-6 protein levels in plasma don’t show a significant change overtime**.** Values are means ± SD.

**Supplementary Table S3.** ROS production, TAC, TBARS, 8-isoPGF2α, 8-OHdG and PC concentrations at baseline and during exposure to 3830 m reveal a marked effect of hypobaric hypoxia on cellular OxS homeostasis. BL, baseline; ROS, reactive oxygen species; TAC, total antioxidant capacity; TBARS, thiobarbituric acid-reactive substances; 8-isoPGF2α, 8-isoprostane; 8-OHdG, 8-hydroxy-2' –deoxyguanosine; PC, Protein Carbonyls. Data are shown as mean (± SD). **Value at BL taken from an average population.


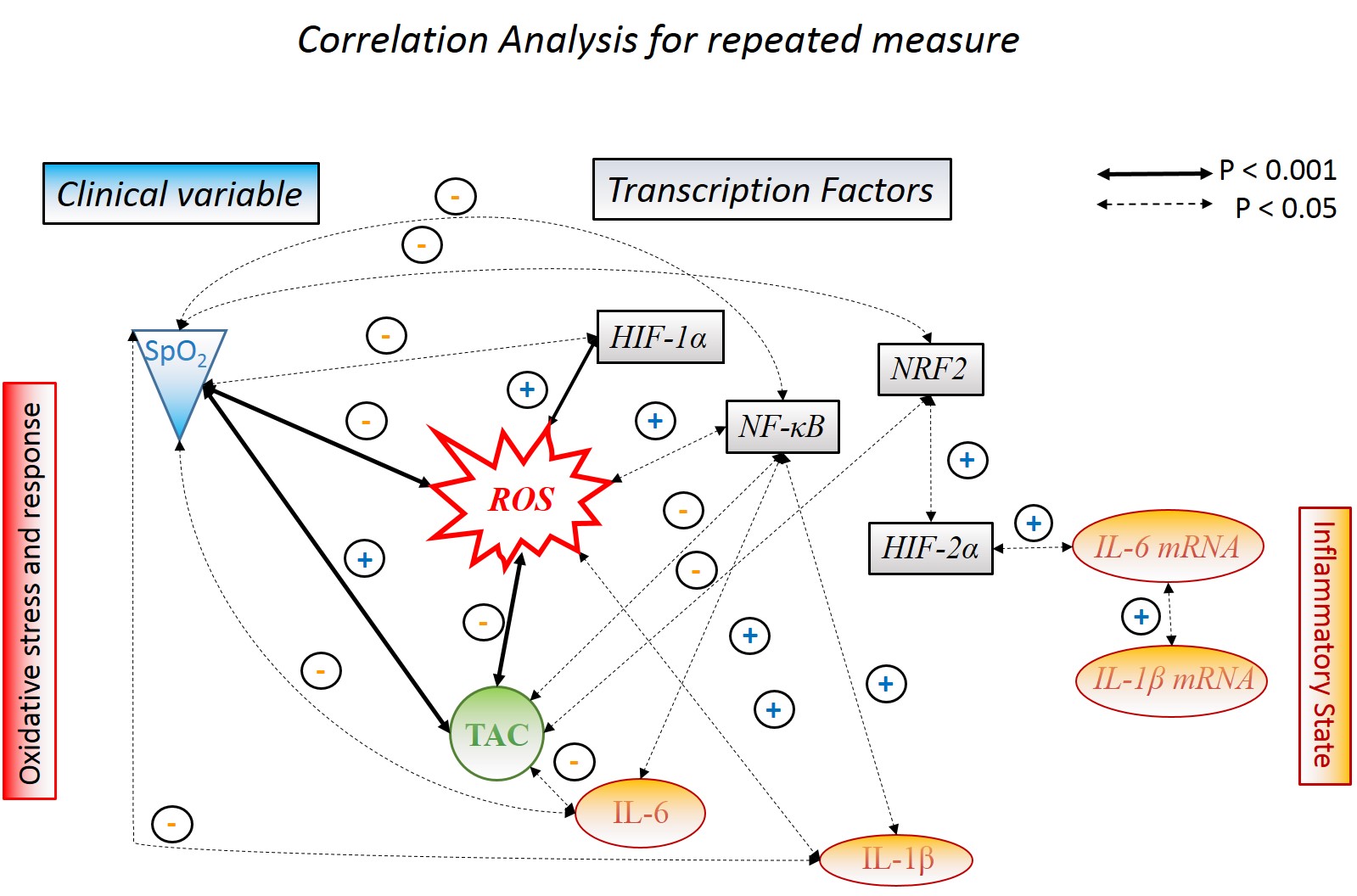


**Supplementary Figure S1.** The proposed scheme highlights marked significant correlation results among all the variables considered in the study and performed by the repeated measures analysis. Strong correlations are showed between the clinical variable SpO_2_, and OxS variables (ROS and TAC) overtime. Weak correlations involved TFs, OxS and Inflammatory variables over time.
